# A case report of testicular diffuse large B-cell malignant lymphoma with cutaneous metastasis: A rare entity

**DOI:** 10.1016/j.ijscr.2021.106519

**Published:** 2021-10-18

**Authors:** Amine Hermi, Mokhtar Bibi, Moez Rahoui, Beya Chelly, Sami Ben Rhouma, Yassine Nouira

**Affiliations:** a University Tunis Manar, Faculty of Medicine of Tunis, Department of Urology, La Rabta Hospital, Tunis, Tunisia; b University Tunis Manar, Faculty of Medicine of Tunis, Department of Pathology, La Rabta Hospital, Tunis, Tunisia

**Keywords:** Testicular, Lymphoma, Metastasis, Skin, Orchidectomy, Case report

## Abstract

**Introduction and importance:**

Primary testicular lymphoma (PTL) is a variety of extra-nodal lymphoma taking origin from testis. It accounts 5% of all testicular tumors. Metastasis may occur in contralateral testis, bone, central nervous system and rarely in skin. Herein, we present the case of testicular diffuse large B-cell malignant lymphoma with cutaneous metastasis.

**Case presentation:**

A 60-year-old male presented with swollen painless solid right testis, with homolateral inguinal nodes. Testicular tumors markers were within normal range. Right radical orchidectomy was performed. Histopathological examination concluded to the diagnosis of Diffuse Large B Cell Lymphoma. Four weeks later, the patient presented alteration of general condition and multiples cutaneous centimetric lesions located in the right inguinal region. Biopsy of this lesion confirmed the diagnosis of metastases from the testicular lymphoma. The patient deceased three days later, before starting further treatment.

**Clinical discussion:**

Primary testicular lymphoma is a rare variety of testicular tumors. The prognosis is poor. Metastasis may occur in different sites such as contralateral testis, central nervous system, and skin. The prognosis is usually poor in the rare case of cutaneous metastasis.

**Conclusion:**

Primary testicular tumor is an aggressive rare variety of testicular tumors with poor prognosis. Cutaneous metastasis is rarely reported. Cutaneous lesions should be explored and suspected to be malignant. Early treatment with rapid multidisciplinary management is the key for adequate approach.

## Highlights

• Primary testicular lymphoma is rare, aggressive testicular tumor.
• Skin metastasis is extremely rare, and if present, compromises the prognosis.
• Diagnosis may be comforted by 18F-PET-CT.
• Orchidectomy followed by chemotherapy is the recommended therapeutic options.

## 1 Introduction

Primary testicular lymphoma (PTL) is a variety of extra-nodal lymphoma taking origin from testis. It accounts 5% of all testicular tumors, with predominance of the histological sub-type of diffuse large B-cell lymphomas (DLBCL) [1]. Metastasis may occur in contralateral testis, bone, central nervous system and rarely in skin. Herein, we present the case of testicular diffuse large B-cell malignant lymphoma with cutaneous metastasis. This work is reported in line with the SCARE 2020 criteria [2].

## 2 Presentation of case

A 60-year-old patient, with no personal or family medical history, was referred to our department for a swollen painless right testis, progressively evolving since 3 months. No associated symptoms were reported (sweating or weight loss).

On clinical examination, the patient was not febrile. A painless solid right testicular mass, was found, inseparable from testis. Left testis was normal. Enlarged centimetric right inguinal lymph nodes were palpable. No other lymph nodes were detected elsewhere.

Laboratory investigations revealed hyperleukocytosis with neutrophilia and normal lymphocyte count. Testicular tumor markers including carcinoembryonic antigen, lactate dehydrogenase and human chorionic gonadotropin were within normal range (3 μg/L, 200 UI/L and 20 pg/mL, respectively).

Scrotal ultrasonography was performed and showed heterogenous enlarged right testis tissue with multiple hypoechogeneous areas. Doppler ultrasound showed increased flow within enlarged testicular tissue. An associated minim hydrocele was also described. No anomalies were noted in left testis.

The patient underwent right radical orchidectomy performed by an experienced professor urologist surgeon. Dissection of the spermatic cord was difficult because of the extended tumoral infiltration of the ladder, and large regional lymph nodes found per operatively.

**Fig. 1:**
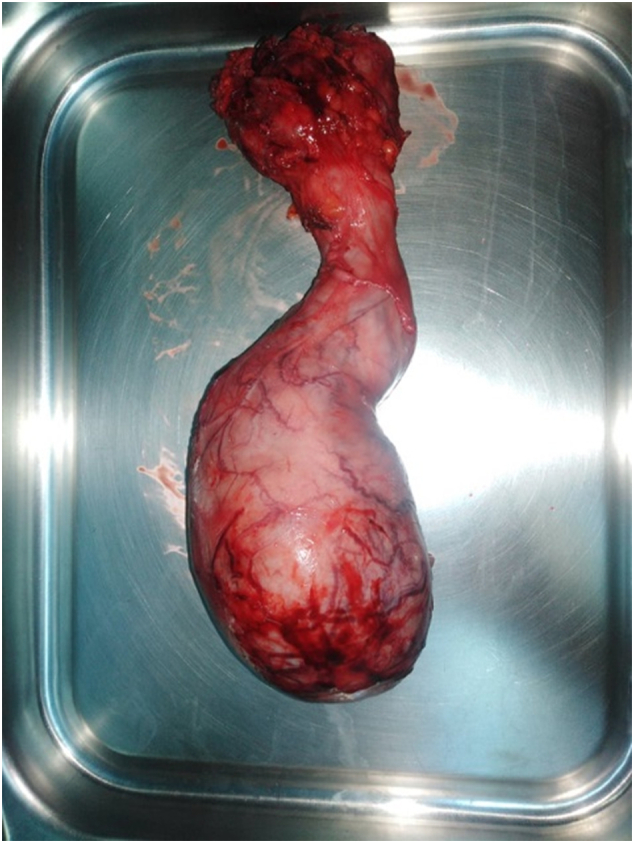
Operative specimens of radical orchidectomy showing infiltration of the testis and the spermatic cord.

On gross examination, the operative specimens measured 110 × 70 × 60 mm. The spermatic cord and the testis were almost entirely replaced by yellowish-white necrosis and fleshy cut surface process Fig. 1.

**Fig. 2:**
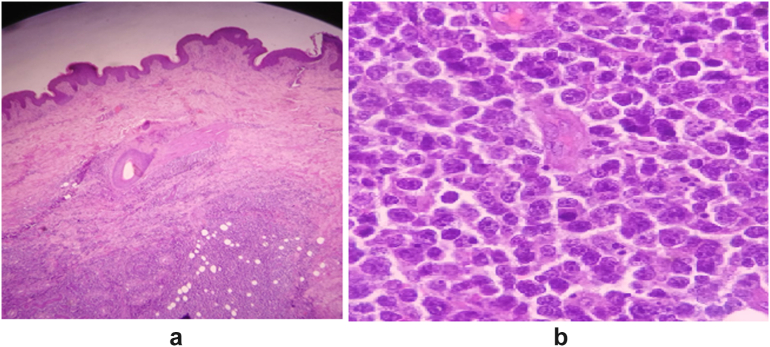
2A. Dermis showing dense proliferation of atypical lymphoid cells (Hematoxylin and eosine (HE 40×)). 2B. Atypical lymphoid cells of medium to large size with frequent mistoses, and irregular shaped nuclei with prominent nucleoli (HE 400×).

Histopathological examination concluded to the diagnosis of diffuse large B cell lymphoma (DLBCL), based on the morphological and immunohistochemical features Fig. 2A,B.

The postoperative course was uneventful. The patient was released, 2 days after surgery, and referred to begin chemotherapy.

**Fig. 3:**
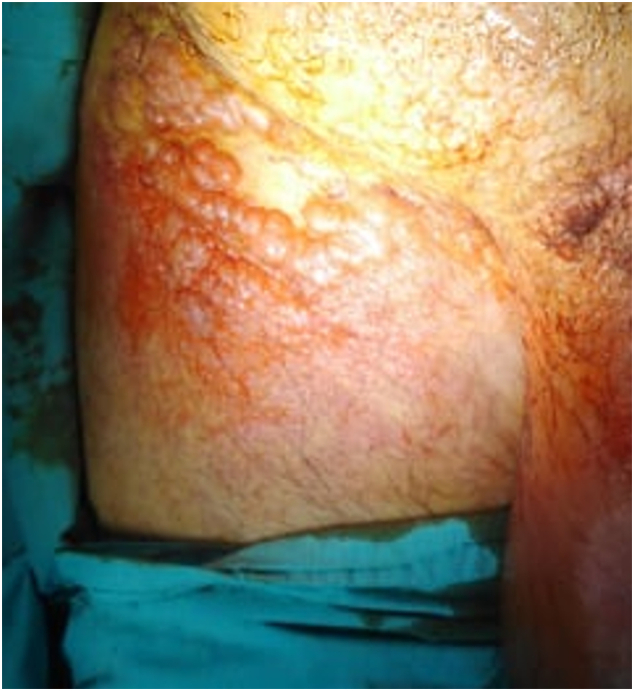
Cutaneous lesions in the right inguinal region, suspected to be PTL metastasis.

The patient presented in our outpatient department, 4 weeks later, with alteration of general condition. Chemotherapy was not started yet. Physical examination revealed centimetric multiple skin lesions with variable size located in the right inguinal region. These cutaneous lesions were suspected to be metastases from the testicular lymphoma Fig. 3.

Biopsy of these lesions confirmed this diagnosis with a morphology and immunohistochemical profile similar to that of the testicular tumor.

**Fig. 4:**
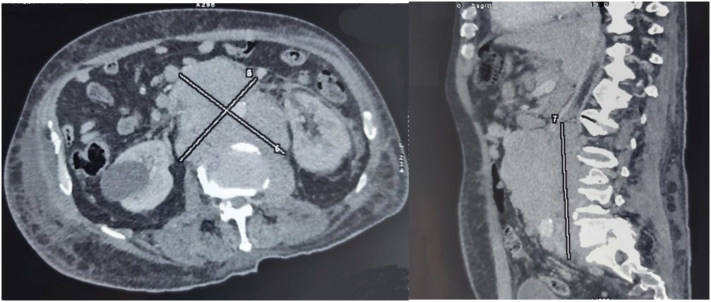
Voluminous retroperitoneal lymph nodes in transversal and sagittal views of thoraco-abdomino-pelvic computed tomography, realised 4 weeks after orchidetomy.

Thoraco-abdominopelvic computed tomography scan was performed and showed multiple and diffuse enlarged lymph nodes located in the retroperitoneum, mesenterium and lesser omentum. Large lomboaortic nodes measuring 120 × 104 × 152 mm, were described with extended thrombosis of the inferior vena cava Fig. 4.

The patient deceased after three days, before starting further treatment.

## 3 Discussion

Testis is an uncommon site of extranodal lymphoma is a rare entity with annual incidence of 0.26 cases per 100,000 person-years [1]. The median age at diagnosis reported in literature is 67 years old [3]. It occurs rarely in children. While PTL accounts for less than 2% of all non-Hodgkin lymphomas (NHL) and approximately 5% of all testicular neoplasms [3], it is the most prevalent histological type of testicular cancer arising in men older than 60 years of age.

The most frequent presenting symptom of PTL is a unilateral testicular mass of variable size [4].

Of all testicular tumors, PTL is stated to be the most common bilateral type, approaching 40% in large series [3], [5] with asynchronous involvement in 37% of cases [5]. Contiguous invasion of the epididymis and the spermatic cord can be seen in 60% and 39% respectively [5].

This diagnosis of PTL is confirmed through orchidectomy or testis biopsy [1]. The predominant histological subtype of PTL is diffuse large B-cell lymphoma, comprising 80% of cases [3].

PTL may be misdiagnosed as a seminoma or embryonal carcinoma [5]. Age at diagnosis, immunohistochemistry and morphological features, help to pinpoint the correct diagnosis.

PTL presents clear extranodal tropism. It mainly infiltrates the contralateral testis and the central nervous system. Other sites can be reached such as skin, adrenal, lung, gastrointestinal, kidney, bone and other soft tissue [1], [4].

Skin metastases occur in 6% to 13% of testicular lymphoma cases [5]. It is believed that it occurs by lymphatic dissemination, which may explain the tendency of the cutaneous lesions to be close to the primary site of the tumor [5]. Our patient presented with skin secondary lesions of the inguinal region ipsilateral to the testicular mass. Generally, PTL is an extremely aggressive malignant tumor with poor progression-free survival (PFS) and overall survival (OS). Although orchiectomy is indicated for both diagnostic and therapeutic purposes, prognosis is considered to be poor in patients treated only with orchiectomy [3].

At present, a standard treatment for PTL has yet to be established, given the rarity of this disease and by the absence of prospective randomized controlled trials available.

Treatment of PTL has generally included orchiectomy, followed by systematic doxorubicin based chemotherapy (CHOP), prophylactic radiation of contralateral testis and prophylaxis intrathecal chemotherapy. Many recent studies emphasis an increase of 10 to 15% in survival with the incorporation of Rituximab into standard therapy with CHOP (R-CHOP21) with minimal added toxicity, in both limited and advanced stage disease. The benefit of prophylactic radiation to reduce the risk of contralateral testis relapse has been confirmedly demonstrated in some studies.

Due to the aggressive nature and rapid progression of testicular DLBCL, timely and correct diagnosis is of importance for the start of treatment. 18F-fluorodeoxyglucose positron emission tomography with computed tomography (18F-FDG PET-CT) is of great value in evaluating skin lesions malignancy and for the identification of malignant lymphomas, with however rare false-positive findings from physiological uptake and infective or inflammatory conditions [6].

## 4 Conclusion

PTL is an aggressive form of extra-nodal lymphoma, with poor prognosis. Cutaneous metastasis is extremely rare and compromises the prognosis. Early diagnosis and no delay in multidisciplinary approach are the keywords for adequate management of this disease.

## Sources of funding

This research did not receive any specific grant from funding agencies in the public, commercial, or not-for-profit sectors.

## Ethical approval

N/a.

## Consent

Written informed consent was obtained from the patient for publication of this case report and accompanying images. A copy of the written consent is available for review by the Editor-in-Chief of this journal on request.

## CRediT authorship contribution statement

Amine Hermi: Data collection, Manuscript writing, Results discussion.

Mokhtar Bibi: Manuscript writing and revision.

Moez Rahoui: Manuscript writing and revision.

Beya Chelly: Paper revision.

Sami Ben Rhouma: Paper and figures revision.

Yassine Nouira: Paper revision.

## Guarantor

Amine Hermi is the guarantor of the study and accept full responsibility for the work and/or the conduct of the study, had access to the data and controlled the decision to publish.

## Declaration of competing interest

Authors do not report any conflict of interest
